# Effects of Gamma Radiation-Induced Crosslinking of Collagen Type I Coated Dental Titanium Implants on Osseointegration and Bone Regeneration

**DOI:** 10.3390/ma14123268

**Published:** 2021-06-13

**Authors:** Won-Tak Cho, So-Yeun Kim, Sung-In Jung, Seong-Soo Kang, Se-Eun Kim, Su-Hyun Hwang, Chang-Mo Jeong, Jung-Bo Huh

**Affiliations:** 1 Department of Prosthodontics, Dental Research Institute, Dental and Life Sciences Institute, Education and Research Team for Life Science on Dentistry, School of Dentistry, Pusan National University, Yangsan-si 50612, Korea; joonetak@hanmail.net (W.-T.C.); hsh2942@hanmail.net (S.-H.H.); cmjeong@pusan.ac.kr (C.-M.J.); 2 Department of Prosthodontics, Biomedical Research Institute, Pusan National University Hospital, Busan 49241, Korea; soyeunkim179@gmail.com; 3 Advanced Radiation Technology Institute, Korea Atomic Energy Research Institute, 29 Geumgu-gil, Jeongeup-si 56212, Korea; sijeong@kaeri.re.kr; 4 Department of Veterinary Surgery and R&BD Center, College of Veterinary Medicine, Chonnam National University, Gwangju 61186, Korea; vetkang@chonnam.ac.kr (S.-S.K.); ksevet@chonnam.ac.kr (S.-E.K.)

**Keywords:** bone regeneration, collagen, gamma radiation, surface modification, titanium implant

## Abstract

This study aimed to compare two methods of crosslinking collagen type I on implanted titanium surfaces, that is, using glutaraldehyde (GA) or gamma-rays (GRs), in a beagle dog model. For in vivo experiments, implants were allocated to three groups and applied to mandibular bone defects in beagle dogs; Group SLA; non-treated Sandblasted, large grit, acid-etched (SLA) implants, Group GA; SLA implants coated with GA crosslinked collagen type I, Group GR; SLA surface implants coated with collagen type I and crosslinked using 25 kGy of ^60^Co gamma radiation. New bone μCT volumes were obtained, and histologic and histometric analyses were performed in regions of interest. The GR group had significantly better new bone areas (NBAs) and bone to implant contact (BIC) results than the SLA group (*p* < 0.05), but the GA and GR groups were similar in this respect. New bone volumes and inter-thread bone densities (ITBD) were non-significantly different in the three groups (*p* > 0.05). Within the limits of this study, gamma-ray collagen crosslinking on titanium implants can be considered a substitute for glutaraldehyde crosslinking.

## 1. Introduction

The interaction between bone and implant interfaces is the key to osseointegration, and various methods of modifying the surfaces of titanium implants have been introduced to improve this process [1,2,3]. Ti surface modifications influence bone regeneration and biocompatibility and facilitate successful implant fixation without soft tissue intervention [4,5,6]. Increasing surface roughness and coating implants with biocompatible materials or growth factors are known to increase the osseointegration of Ti implants [7]. In particular, collagen type I is used as a biocompatible polymer because it promotes osteoblast differentiation and provides a suitable environment for bone formation [8,9,10].

At the molecular level, collagen type I has a tangled, triple-helix structure with two α1 (I) and one α2 (I) polypeptide chains, and many years of clinical use have proven it to be a biocompatible, bioactive, bioresorbable material [11,12]. Implant surfaces coated with crosslinked collagen type I provide a favorable environment for initial osteoblast adhesion and stimulate their proliferation [9]. However, rapid absorption and decomposition by enzymes and immune reactions against animal-derived collagen cause type I collagen degradation; therefore, crosslinking is required to improve its in vivo stability [13]. Glutaraldehyde (GA) is commonly used as a crosslinker for collagen-based biomaterials, and GA cross-linking of collagen decreases its antigenicity, makes it resistant to phagocytosis, and invisible to the immune system [9,14,15]. However, like other chemical crosslinking methods, GA has been reported to produce harmful cytotoxic residues and increase proinflammatory cytokine release by macrophages [16,17,18,19]. Recently, different types of irradiation-induced crosslinking methods such as gamma-ray and ultraviolet have been used in preference to chemical crosslinkers substances to crosslink polymers like collagen [13,20,21].

Unlike ethylene oxide or GA sterilization, gamma radiation leaves no harmful residues that could potentially harm human health or the environment and is used to sterilize medical devices [6,22]. Moreover, gamma radiation-induced polymer crosslinking enables control of radiation-induced decomposition reactions, e.g., polymer chain scission, which can cause molecular weight reductions, as its effects are not dependent on material compositions [23,24,25]. Furthermore, when collagen is irradiated with gamma rays, peptide bonds are destroyed due to amino acid deformation, and hydrophilicity is improved by hydrogen bond formation [26]. In addition, enhancements of sandblasted, large grit, acid-etched (SLA) implant surface hydrophilicity have been reported to increase alkaline phosphatase (ALP) by more than 2-fold in cell culture experiments [27].

A previous comparative study concluded that there was no difference between the cytotoxicities of the gamma radiation crosslinked group and a GA-crosslinked group, based on absorbance data. However, gamma crosslinked collagen-coated Ti implants had significantly higher BICs than non-coated controls in a small animal model [28]. Therefore, we compared the effects of GA and gamma-ray crosslinking of collagen type I on the surfaces of SLA Ti implants in a beagle model to determine the effectiveness of gamma-induced cross-linking. The null hypothesis was that bone regeneration and osseointegration after GA or gamma crosslinking of collagen type I coated SLA implants are similar.

## 2. Materials and Methods

### 2.1. Experimental Materials

Collagen type I solution (0.5% (*w*/*v*)) was obtained by dissolving collagen (source: porcine skin, atelocollagen type I, Matrixen-PSP, Sk Bioland Co. Ltd., Cheonan, Korea) in 0.05 M acetic acid (Sigma-Aldrich, St. Louis, MO, USA) at room temperature. The Ti implant fixtures (D 4.0 mm × H 8.0 mm, SLA surface, Cowellmedi Co., Ltd., Pusan, Korea) were placed in a 0.5% (*w*/*v*) collagen type I solution. Bubbles on implant surfaces were removed by sonication (Elmasonic, S 180 H, Elma Schmidbauer, Elma, Germany) for 10 min. Then implants were placed in climate chambers (MIR-253, SANYO, Moriguchi, Japan) to dry for 1 h at 4 ℃. Implants in the GA group were crosslinked by placing them in 2.5% (*v*/*v*) GA (DAEMYUNG CHEMICAL, Gyeonggi-do, Korea) for 1 h. Unreacted GA and collagen type I were then removed by washing in distilled water, dried in a vacuum oven (WOV-30, DAIHAN Scientific Co.Ltd., Gangwon-do, Korea) for 3 days [28], and sterilized with ethylene oxide (Manufacturer, City, State, Country). The implants of the GR (gamma-radiation) group were immersed in collagen solution in the same way as in the GA group, followed by ultrasonic cleaning for 10 min, and dried in a climate chamber for 1 h. The GR group implants were then irradiated with ^60^Co gamma rays (MDS Nordion, Ottawa, ON, Canada) at 25 kGy for 1 h [28].

### 2.2. In Vitro Study

#### 2.2.1. Scanning Electron Microscopy (SEM) Analysis

Surface images of implants were obtained using an SEM unit (Hitachi S3500N, Hitachi, Tokyo, Japan) at magnifications of ×40, ×5000, and ×50,000. For the SEM study, implants were splutter-coated with gold (SCD 005, BAL-TEC, Balzers, Liechtenstein). SEM images were obtained at 15 kV.

#### 2.2.2. X-ray Photoelectron Spectroscopy (XPS)

Implant surfaces were analyzed by XPS (AXIS SUPRA, Kratos Analytical Ltd., Manchester, UK) using a monochromatic Al-Kα (1486.6 eV) X-ray source (1486.6 eV) at 15 kV and 225 W. The binding energy scale was calibrated at the C 1s level (284.5 eV). Implants in each group were subjected to a compositional survey at a pass energy of 160 eV, and core level spectra were obtained at a pass energy of 20 eV. Data analysis was performed using data reduction software (Vision 1.5, Kratos Analytical Ltd., Manchester, UK). Deconvoluted spectra were fitted using a Gaussian−Lorentzian sum function (20% Gaussian and 80% Lorentzian) using XPSPEAK Version 4.1 (Dr. Raymond Kwok, Hong Kong, China).

### 2.3. In Vivo Experiment

#### 2.3.1. Experimental Animals

This study was approved by the Ethics Committee on Animal Experimentation of Chonnam National University (CNU IACUC-YB-2018-94). Six beagles (males, three years old, 12 kg) were used in the study.

#### 2.3.2. Surgical Procedure

Beagles were anesthetized with a medetomidine (Tomidin^®^, Provet, Istanbul, Turkey) 10 μg/kg and tiletamine-zolazepam (Zoletil 50^®^, Virbac Laboratories, Carros, France) at 5 mg before the procedure and followed by isoflurane inhalation anesthesia (Sevoflurane^®^, Hana Pharm Co., Seoul, Korea). Anesthesia was maintained using tramadol (Maritrol^®^, Cheil Pharmaceutical, Uiwang, Korea) 2 mg/kg and carprofen (Rimadyl^®^ inj, Zoetis, Parsippany, NJ, USA) 2.2 mg/kg IV. In addition, infiltration anesthesia at surgical sites was performed using 0.4 mL bupivacaine (Bupivacaine HCl 0.5% Inj., Myungmoon Pharm Co., Seoul, Korea). To prevent infection, 20 mg/kg of cefazolin sodium (Cefazolin^®^, Chongkundang Pharm Co., Seoul, Korea) was injected subcutaneously.

**Figure 1:**
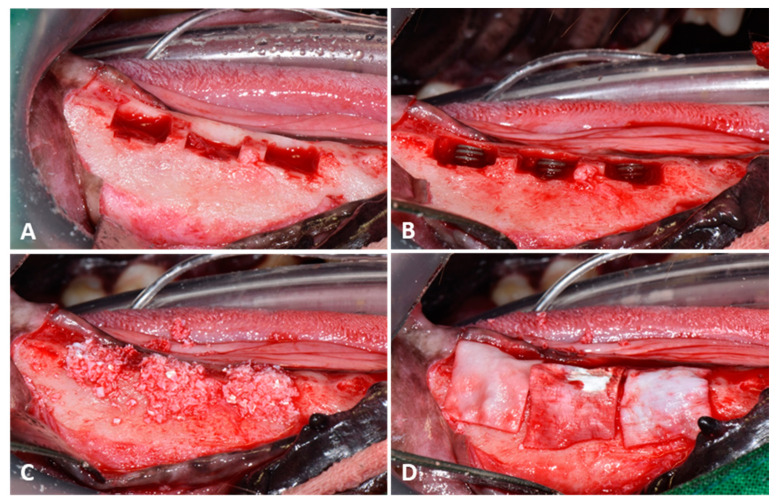
Surgical procedures used to place surface-treated implants in beagle mandibles. (**A**) Creation of buccal cubic defects, (**B**) Implant placement, (**C**) Distribution of bone graft material, (**D**) Collagen membrane placement.

Mandibular premolars (P1–P4) and M1 molar were extracted after full mouth scaling. Implants were placed after extraction sites had healed for 8 weeks [28,29]. General anesthesia and local infiltration anesthesia were applied as described for extractions. A mid-crestal incision was made at each premolar site, and vertical incisions were made at the mucogingival junction. After mucoperiosteal flap elevation, crestal bone was homogenized by osteoplasty using a bone file and rongeur. Buccal cuboid defects, approximately 5 mm in height from crestal bone, 5 mm deep from the surface of the buccal bone, and 8 mm in width mesiodistally, were created using a straight fissure carbide bur under saline irrigation (JW Pharmaceutical Co. Ltd., Gyeonggi-do, Korea) (Figure 1A). Animals were allocated randomly to the three study groups, which were as follows:

- Group SLA (*n* = 12): Non-treated SLA implants.
- Group GA (*n* = 12): SLA implants coated with GA crosslinked collagen type I.
- Group GR (*n* = 12): SLA implants coated with 25 kGy ^60^Co gamma radiation crosslinked collagen type I.

Then, 36 implants (Cowell Medi Co, Ltd., Busan, Korea), 4 mm in diameter and 8 mm high, were implanted in the mandibular defects of 6 animals to expose three threads (Figure 1B). Peri-implant defect sites were grafted with porcine xenografts (Bone-XP, MedPark, Busan, Korea) (Figure 1C), and bone regeneration was guided using resorbable collagen membrane (Bone-D, MedPark, Busan, Korea) (Figure 1D). Surgical sites were sutured with 4-0 Vicryl (Mersilk, Ethicon Co., Livingston, UK). Post-operative care consisted of oral amoxicillin-clavulanate (Amocla^®^, Kuhnil Pharm Co., Seoul, Korea) 12.5 mg/kg, firocoxib (Previcox, Merial, France) 5 mg/kg, and famotidine (Famotidine^®^, Nelson, Seoul, Korea) at 0.5 mg/kg for 2 weeks.

Eight weeks after implant placements, animals were sacrificed by potassium chloride intravenous injection (JW Pharmaceutical Co. Ltd., Gyeonggi-do, Korea) under general anesthesia, and mandibular bones were harvested and fixed in neutral buffered formalin (Duksan Pure Chemical. Co. Ltd, Gyeonggi-do, Korea) for 2 weeks.

#### 2.3.3. Micro-Computed Tomography (µCT) Analysis

**Figure 2:**
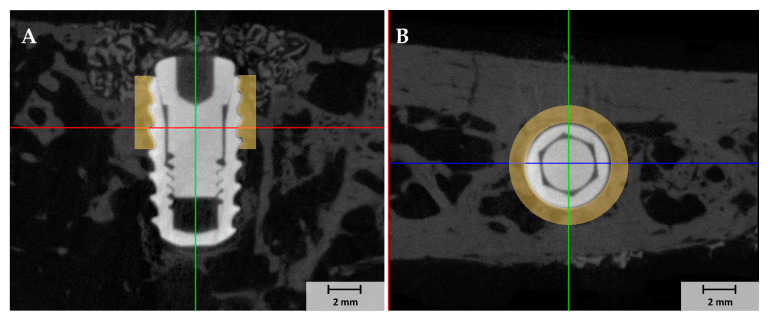
The µCT images of regions of interest (ROIs) which included 1 mm around each implant. (**A**) buccal view, (**B**) occlusal view.

Mandibles were wrapped with Parafilm M^®^ (Heathrow Scientific, Vernon Hills, IL, USA) and scanned by µCT (Skyscan-1173, ver. 1.6, Bruker-CT Co., Kontich, Belgium) at 130 kV and an intensity of 60 µA to obtain the µCT images of regions of interest (ROIs). We used a pixel resolution of 24.15 µm to determine new bone volumes (NBVs) in defect areas around implants. µCT image reconstructions were performed using Nrecon reconstruction software ver. 1.7.0.4 (Bruker-CT Co., Kotich, Belgium). The study used 1 mm diameter ROIs around implants (Figure 2).

#### 2.3.4. Histologic Analysis

**Figure 3:**
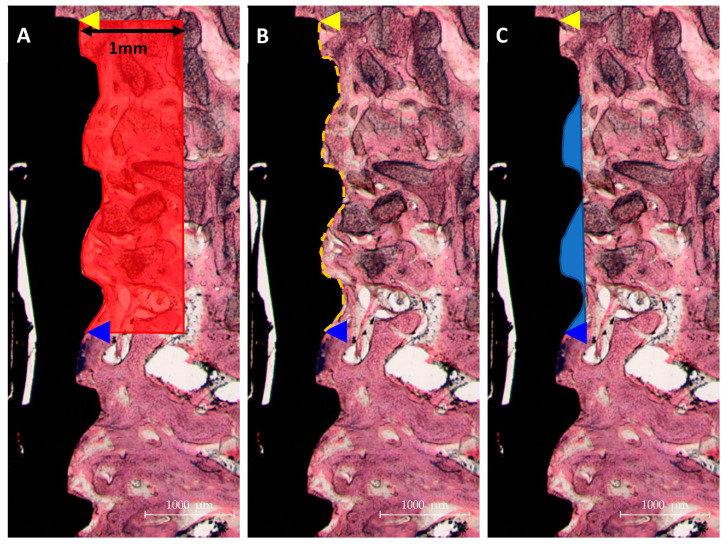
Histometric measurements in regions of interest (ROIs). ROIs were fixed from implant platforms to the third thread and at 1 mm around implants in occlusal view. (**A**) NBA: New bone area, (**B**) BIC: Bone-to-implant contact, (**C**) ITBD: Inter-thread bone density.

After µCT analysis, mandibular bone specimens were dehydrated in an ethanol series (Duksan Pure Chemical. Co. Ltd, Gyeonggi-do, Korea) 70, 80, 90, and 100%, infiltrated with resin (Technovit 7200, Heraeus KULZER, Hanau, Germany) for a week, fixed to an embedding frame, and embedded using a UV curing system (KULZER EXAKT 520, Heraeus Kulzer, Norderstedt, Germany). Polymerized specimens were sectioned at 400 µm at implant centers using a diamond cutter (KULZER EXAKT 300 CP Band System, Exakt Apparatebau, Norderstedt, Germany). Then, they were polished to a thickness of 30 µm using an EXAKT grinding machine (KULZER EXAKT 400CS, Exakt Apparatebau, Norderstedt, Germany), mounted on slides, and stained with hematoxylin and eosin (H&E). Images of stained specimens were obtained using a light microscope (Olympus BX, Olympus, Tokyo, Japan). BIC and ITBD values and new bone areas (NBAs) were measured using an image analysis program (ver. 7.5, i-solution, IMT i-solution. Inc., Vancouver, BC, Canada) by a trained investigator (Figure 3). ROIs were set at exposed three upper threads and 1 mm around fixtures, as shown in Figure 2.

NBAs (%) = New bone area (mm^2^)/Total ROI area (mm^2^) × 100 (1)

BIC (%) = Length of the new bone to implant contact (mm^2^)/Total ROI length of implant (mm^2^) × 100 (2)

ITBDs (%) = New bone area of inter thread (mm^2^)/Total area of inter thread (mm^2^) × 100 (3)

#### 2.3.5. Statistical Analysis

Results are presented as means ± standard deviations (SDs), and the analysis was performed using SPSS Ver. 25 (SPSS Inc., Chicago, IL, USA). Since NBAs, ITBDs, NBV, and BIC values were not normally distributed by the normality test, the Kruskal-Wallis one-way analysis was used to determine the significances of intergroup differences. The Mann-Whitney U test was applied as a post hoc test. Statistical significance was accepted for *p* values < 0.05.

## 3. Results

### 3.1. In-Vitro Study

#### 3.1.1. Collagen Crosslinked Ti Implant Surface Morphologies

**Figure 4:**
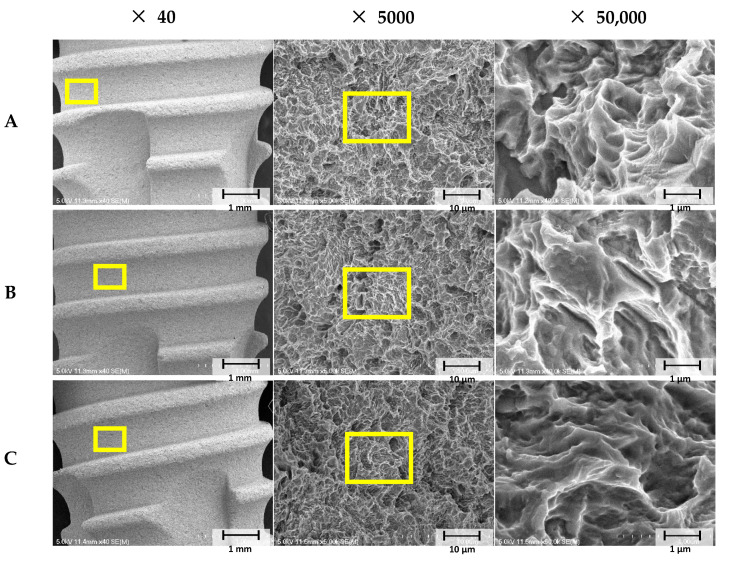
The scanning electron microscopy (SEM) images. (**A**) The SLA (Sandblasted, large grit, acid-etched implant surface) group, (**B**) The GA (glutaraldehyde) group, and (**C**) the GR (gamma-radiation) group. [Original magnifications: ×40, ×5000, and ×50,000].

When collagen was crosslinked using GA or 25 kGy Gamma rays on SLA implant surfaces, surface morphologies were similar due to their rough SLA surfaces (Figure 4).

#### 3.1.2. XPS Findings

**Figure 5:**
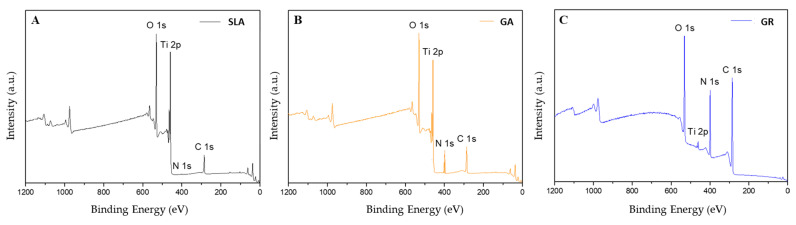
Surface XPS spectra of the three study groups. (**A**) The SLA group, (**B**) the GA group, and (**C**) the GR group.

Surface elemental compositions were determined by XPS (Figure 5).

The SLA group had the lowest nitrogen content (0.33%), followed by the GA group (6.22%) and the GR group (17.64%). Since the major component of collagen is gelatin (a protein), a large amount of nitrogen indicates good crosslinking [30] (Table 1).

### 3.2. In Vivo Study

#### 3.2.1. Clinical Findings

All beagles survived the surgical procedures without complications, such as inflammation or infection. Mandibular jaw segments were harvested after sacrifice.

#### 3.2.2. Micro-Computed Tomography (µCT) Findings

**Figure 6:**
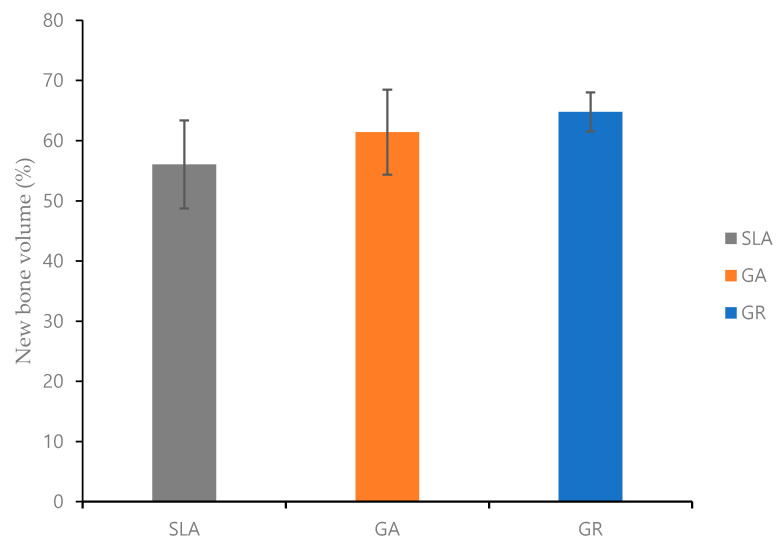
The volumetric ROI analysis of new bone.

In regions of interest, NBV was 64.78 ± 3.24% in the GR group, 61.42 ± 7.07% in the GA group, and 56.06 ± 7.31% in the SLA group. Thus, although NBV was relatively high in the GR group, differences were not significant (Figure 6).

#### 3.2.3. Histological Findings

**Figure 7:**
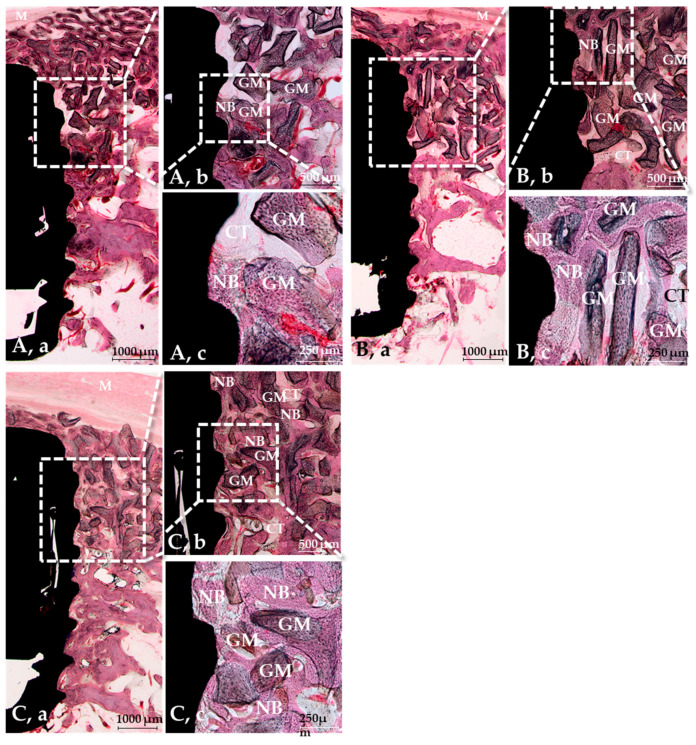
H&E stained sections at 8 weeks post-implantation. (**A**) The SLA group, (**B**) the GA group, (**C**) the GR group, (**a**) ×12.5, (**b**) ×40, (**c**) ×100. Note: NB = New bone, GM = Bone graft material, CT = Connective tissue, M = Membrane.

The histological results of the SLA, GA, and GR groups are shown in Figure 7. No abnormal inflammatory cells or singularities were found in any group. However, new bone formation was observed between the third and second threads in the SLA group but distributed evenly in all the GA and GR groups. The crosslinked groups exhibited more new bone formation than the SLA group, but the new bone formation was similar in the GA and GR groups.

#### 3.2.4. Histometric Findings

**Figure 8:**
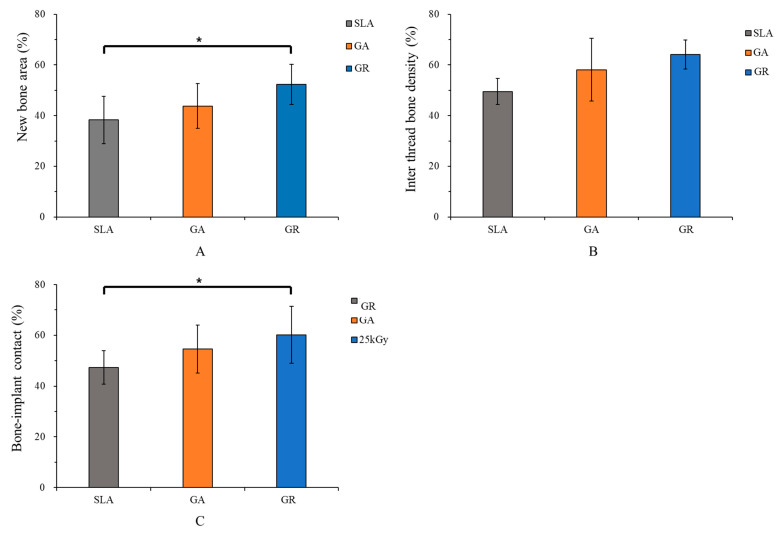
Histometric analysis within regions of interest (ROI). (**A**) New bone area (%), (**B**) Inter thread bone density (%), (**C**) Bone-implant contact (%). * Indicates statistical significance (*p* < 0.05).

Histometric results are summarized in Table 2 and Figure 8. NBA values of the SLA, GA, and GR groups were 38.27 ± 9.34%, 52.37 ± 7.93%, and 43.77 ± 8.81%, respectively, and were significantly higher in the GR group than in the SLA group (*p* < 0.05).

ITBD results of the SLA, GA, and GR groups were 49.52 ± 5.11%, 58.10 ± 12.33%, and 64.10 ± 5.65%, respectively, and no significant intergroup difference was found (*p* > 0.05).

Corresponding BIC results were 47.3 ± 6.58%, 54.61 ± 9.4%, and 60.19 ± 11.23%, and BIC was significantly greater in the GR group than in the SLA group (*p* < 0.05). On the other hand, the results of the GR group were similar to the values of the GA group in NBA, ITBD, and BIC (*p* > 0.05).

## 4. Discussion

Commercially available dental implants are generally considered to have high biocompatibility and surfaces suitable for bone regeneration [4,31], and this is supported by the results of prospective and retrospective clinical studies, which reported implant 10-year survival rates exceeding 90% [32,33,34,35]. Nevertheless, dental implant failure due to osseointegration failure often occurs unexpectedly and remains an important clinical problem [36,37]. Therefore, studies on implant surface modification methods have also been conducted to improve osseointegration using surface treatments and collagen as bioactive material [38,39]. However, extracted collagen’s mechanical properties and stabilities are inferior; thus, its potential is limited [40,41]. GA has been used as a collagen crosslinking agent for several decades, but some GA probably remains in situ after crosslinking. Protocols for removing unreacted GA have been proposed to solve this problem, but unfortunately, these methods have also been reported to have cytotoxic side effects [42,43]. On the other hand, gamma-ray-based crosslinking does not leave harmful residues and has recently been used to crosslink polymers, including collagen [6]. Therefore, this study was conducted to evaluate and compare the merits of crosslinking collagen type I on the surfaces of SLA implants with gamma-ray radiation or GA in a large animal model.

Collagen type I is a useful biopolymer and widely used clinically due to its low immunogenicity, biocompatibility, and biomedical potential [42]. In addition, collagen is known to promote osteoblast adhesion when coated on implant surfaces [9]. Previous in vivo studies have confirmed that collagen treatment promotes bone regeneration following implantation of crosslinked collagen-coated Ti implants and that collagen treatment enhances bone to implant adhesion to bone and accelerates bone formation [44,45]. Likewise, in the present study, NBAs and BIC values were higher in the GR group than in the SLA group, similar to the GA and SLA groups, which suggests 25 kGy gamma-ray exposure provides better crosslinking than GA. Furthermore, XPS analysis showed surface nitrogen levels (17.64%) were higher in the GR group than in the GA group (6.22%). However, the GR group did not significantly differ compared to the GA group (*p* > 0.05).

After machining Ti, its surface is contaminated by adsorbed organic entities such as atmospheric hydrocarbons, water, or cleaning fluids [46,47]. Previous studies that analyzed the chemical compositions of different implant surfaces by XPS have reported carbon deposition percentages ranging from 17.9 to 76.5% [48]. Therefore, gamma irradiation at 25 to 35 kGy has been recommended for the rapid disinfection and sterilization of medical devices. Ueno et al. [49] found that deposited hydrocarbons can be removed by high-energy UV or gamma radiation and that the removal of hydrocarbons improves Ti biocompatibility and induces osseointegration. Our XPS results returned surface carbon figures in the GR, GA, and SLA groups of 0.93, 6.22, and 20.96%, respectively, suggesting that surface carbon was removed by gamma irradiation [30]. This observed reduction in surface carbon levels by gamma irradiation is consistent with the results of previous studies [6].

Accordingly, the present study suggests that gamma irradiation-induced collagen crosslinking enhances Ti implant biocompatibility and bone adhesion in beagle mandible models. Collagen cross-linked implants using gamma irradiation may improve the osseointegration in adverse circumstances requiring transcrestal sinus lift procedures [50]. Besides, in patients with a history of systemic disease, increased implant-bone osseointegration may be an important factor for long-term implant survival [51]. Meanwhile, Misch [52] recommended that the occlusal implant area be made small. Since the increased osseointegration increases the mechanical strength of the bone tissue, the occlusion of the implant prosthesis can be properly distributed [53].

Furthermore, if a substance that induces a stem cell response, such as rhBMP-2, is attached to the collagen-crosslinked implant with gamma rays, better osteoinductivity can be expected. However, the study was limited by the model used, the number of beagles involved, and its short duration. Furthermore, there was no difference in the histological aspect compared to the GA group. In addition, it is considered necessary to compare it with other biocompatible materials other than collagen. Accordingly, we recommend additional experiments be performed to establish a scientific basis for the clinical effectiveness of crosslinking collagen on Ti implants using gamma radiation.

## 5. Conclusions

This study was conducted to assess the effects of gamma radiation-induced collagen crosslinking on osseointegration and bone regeneration in defect areas around SLA implants. Within the limitations of this study, gamma-ray collagen crosslinking was found to be at least as effective as GA crosslinking in terms of bone regeneration efficacy. According to our results, gamma-radiation can be used to effectively crosslink collagen on implant surfaces and not raise concerns about toxic residues. Additional animal studies are required to determine optimum gamma-radiation dose criteria and to more comprehensively evaluate the effect of irradiation on osseointegration.

## Figures and Tables

**Table 1:** Atomic concentrations (at. %) on implant surfaces as determined by XPS.

Elements	Group		
	SLA	GA	GR
C	20.5 ± 0.33	29.87 ± 0.25	64.77 ± 0.42
O	58.22 ± 0.79	46.64 ± 0.49	16.67 ± 0.09
Ti	20.96 ± 0.36	17.27 ± 0.27	0.93 ± 0.03
N	0.33 ± 0.17	6.22 ± 0.13	17.64 ± 0.30

**Table 2:** Mean values of new bone areas (NBAs), inter-thread bone densities (ITBDs), and bone to implant contacts (BICs) as determined by histometric analysis.

Measurement	Group	Mean ± SD	*p*-Value
NBA (%)	SLA	38.27 ± 9.34	0.033 *
	GA	43.77 ± 8.81	
	GR	52.37 ± 7.93	
ITBD (%)	SLA	49.52 ± 5.11	0.053
	GA	58.10 ± 12.33	
	GR	64.10 ± 5.65	
BIC (%)	SLA	47.3 ± 6.58	0.046 *
	GA	54.61 ± 9.4	
	GR	60.19 ± 11.23	

* Indicates statistical significance (*p* < 0.05).

## Data Availability

Data sharing not applicable.
